# Fatigue Crack Length Sizing Using a Novel Flexible Eddy Current Sensor Array

**DOI:** 10.3390/s151229911

**Published:** 2015-12-21

**Authors:** Ruifang Xie, Dixiang Chen, Mengchun Pan, Wugang Tian, Xuezhong Wu, Weihong Zhou, Ying Tang

**Affiliations:** College of Mechatronics Engineering and Automation, National University of Defense Technology, Changsha 410073, China; chendixiang@163.com (D.C.); pmc_nudt@vip.163.com (M.P.); twg_1978@163.com (W.T.); xzwu@nudt.edu.cn (X.W.); zwhgfkd@163.com (W.Z.); tangyingtiger@163.com (Y.T.)

**Keywords:** flexible eddy current array, crack sizing, quantitative nondestructive evaluation, spatial resolution, high sensitive

## Abstract

The eddy current probe, which is flexible, array typed, highly sensitive and capable of quantitative inspection is one practical requirement in nondestructive testing and also a research hotspot. A novel flexible planar eddy current sensor array for the inspection of microcrack presentation in critical parts of airplanes is developed in this paper. Both exciting and sensing coils are etched on polyimide films using a flexible printed circuit board technique, thus conforming the sensor to complex geometric structures. In order to serve the needs of condition-based maintenance (CBM), the proposed sensor array is comprised of 64 elements. Its spatial resolution is only 0.8 mm, and it is not only sensitive to shallow microcracks, but also capable of sizing the length of fatigue cracks. The details and advantages of our sensor design are introduced. The working principal and the crack responses are analyzed by finite element simulation, with which a crack length sizing algorithm is proposed. Experiments based on standard specimens are implemented to verify the validity of our simulation and the efficiency of the crack length sizing algorithm. Experimental results show that the sensor array is sensitive to microcracks, and is capable of crack length sizing with an accuracy within ±0.2 mm.

## 1. Introduction

The key components of aircraft such as engine blades are under the long-term effects of cyclic loads and thermal loads, suffering the danger of fatigue failure and the resulting risk of disastrous accidents. To ensure safety and prevent accidents, quantitative nondestructive evaluation (QNDE) systems have been proposed and developed against this background. Condition-Based Maintenance (CBM) is one kind of maintenance strategy based on device status evaluation, and the status information is obtained by using sensors and external test equipment. While the crack is small enough, the structure can keep working despite its “illness”. Sometimes the structure must be sifted out, which depends on the size of crack. A reasonable maintenance strategy reduces not only maintenance costs, but also the economic losses due to outage. CBM can ensure the reliability and safety of equipment, reduce the operation and support costs, decrease breakdown maintenance and preventive maintenance tasks, and prevent accidents. QNDE of microdefects is one of the key technologies of CBM, which helps to effectively prevent the generation and spread of various mechanical structural failures and eliminate failure in the embryonic stage.

Eddy Current Non-Destructive Testing (ECNDT) [1,2,3,4] has been widely used in non-destructive testing of critical aircraft components, because of its convenience, speediness and high sensitivity in the detection of metal component problems. Cha *et al.* [5] designed a micro-coil sensor with high detection sensitivity, which was fabricated using MEMS technology, whose dimension was only 400 μm × 380 μm × 35 μm. However due to its small size it had to rely on a motion platform, and the detection efficiency is relatively low. Chen *et al.* [6,7] designed an eddy current probe to test the nuclear power plant pipelines, and to reconstruct and classify defects, but its accuracy is only several millimeters.

Flexible eddy current sensors and arrays [8,9] are becoming a hot research topic, aiming to solve the inspection problems of key components with complex geometry. A Meandering Winding Magnetometer (MWM) sensor array fabricated with flexible printed circuit board (FPCB) technology, has been developed to detect fatigue, corrosion, thermal barrier coatings, stress and so on [10,11]. The MWM sensor was used for monitoring the initiation and propagation of fatigue cracks, with a minimum detectable defect length of 200 μm, and depth of 50 μm, but no quantitative detection application was mentioned. Marchand, *et al.* [8] designed a flexible eddy current array probe comprising 96 elements, which consists of an exciting and sensing micro-coil for examining specimens with complex surfaces. The probe was very sensitive to micro-defects, yet no quantitative detection results were disclosed. Endo, *et al.* [12] designed a flexible array eddy current testing (ECT) probe, and a 12 decibel drop method was used to size fatigue crack length, with a sizing accuracy within ±3 mm.

In addition, other techniques have been used to perform quantitative testing of microcracks. Deng *et al.* [13] designed an impedance sensor film for detecting the initiation and expansion of fatigue cracks. A thin film layer of several tens of nanometers was plated on the surface of targets using an ion sputter plating method. The initiation and expansion of fatigue cracks can be monitored by measuring the impedance of the film, which varies because of fatigue crack initiation or expansion. The crack length can be measured with this method and the error is less than 0.068 mm. However, this method requires a metal film to be sputtered on the surface of the subject material, and a high level of surface cleanliness, which can hardly be achieved in many cases, such as in-service equipment like engine blades. Lim and Soh [14] monitored three stages of fatigue crack using an electro-mechanical impedance (EMI) technique which employed the PZT material as the excitation and vibration sensing sensor and they were able to monitor fatigue remotely with high sensitivity and precision. Nevertheless, the position of cracks is unknown and crack visualization is impossible.

In general, although crack sizing has been achieved by researchers using different non-destructive testing techniques, the following disadvantages still exist [14]: preknown defect locations, automation difficulties, operational outages, equipment dismantling, high cost and labor consumption, lack of precision or reliance on motion platforms, low adaptability to complex surfaces, *etc*.

Therefore, a technology which can detect cracks in their early stages or even before formation and allows quantitative tracking of crack propagation and adoption of appropriate maintenance strategies based on crack type, size, *etc.* is urgently needed. According to a report by Fuchs and Stephens [15], up to 90% of structural failures are caused by fatigue cracks. Extensive experiments [6] have shown that fatigue cracks are characterized by small width and approximately rectangular shapes in crosssection, and they are not substantially bifurcated like stress corrosion cracks; besides, the gap between sections, which is filled with air, is about 10–50 μm, thus the defect area is not conductive. This means that fatigue cracks can be simulated using electro-discharge machined defects (EDM defects). Moreover, the length and depth of natural cracks approximately satisfy a 2:1 relationship, while the width of cracks is usually not of concern. In addition, fatigue cracks are mostly surface or sub-surface cracks. Based on the above facts, this paper present the design of a flexible planar eddy current sensor array for quantitative measurement of fatigue crack length in metal components. The novelty of this paper is that a quantitative algorithm named NCSF based on a novel flexible eddy current array (FECA) is developed for fatigue crack length measurement.

## 2. Sensor Design

Although single element eddy current sensors and multi-element sensor arrays are both used for defect sizing, sensor arrays are more widely used because of their advantages such as higher detection efficiency and spatial resolution, and larger coverage [8,12]. Transmit/receive (T/R) type coil configurations have been proven to provide higher detection sensitivity and better signal-to-noise ratio, and thus have been widely used in eddy current sensors [16]. In addition, from the perspective of the actual inspection needs of key aircraft components, the eddy current sensors should meet the following requirements:

1. Flexibility, fit for the defect detection of complex surfaces;
2. High detection efficiency; no need for motion platforms; *in-situ* and in-service inspection;
3. High sensitivity, allowing detection of microdefects;
4. High spatial resolution; high-precision measurement of crack lengths;
5. No need of preknowing the position and orientation of defects.

**Figure 1:**
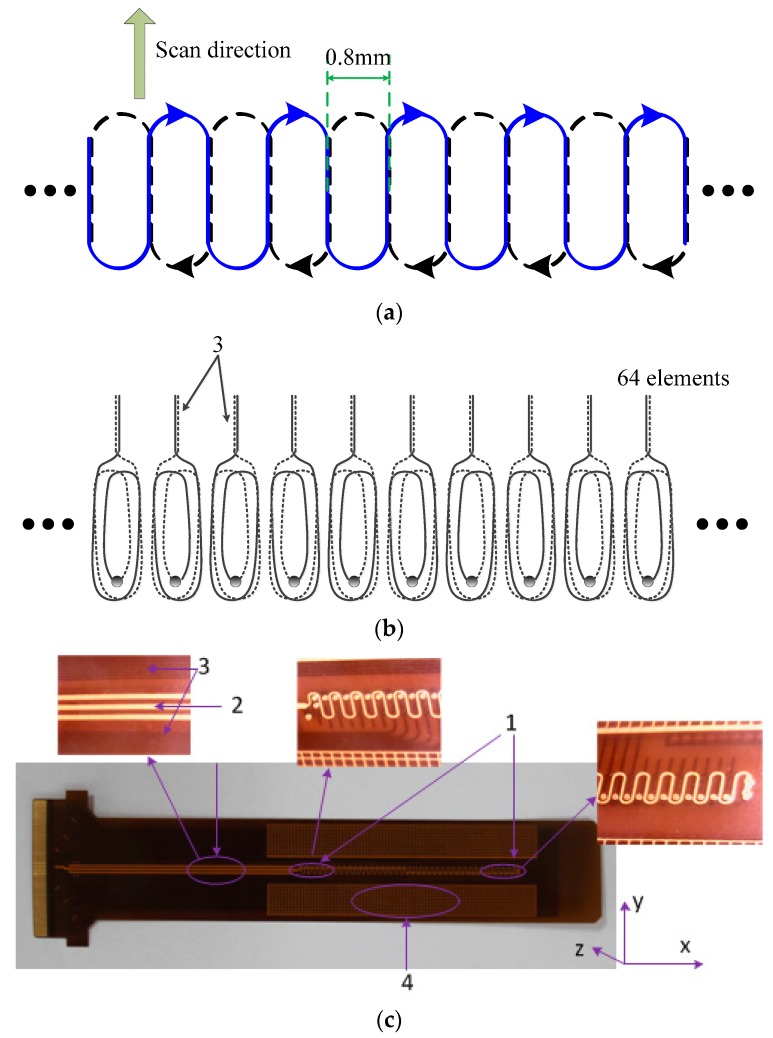
Novel sensor array (**a**) Schematic diagram of the exciting coil; (**b**) Schematic diagram of the sensing coil; (**c**) Actual sensor.

To meet the above requirements, a flexible eddy current sensor array which takes into consideration both sensitivity and resolution and is able to detect microdefects and measure their lengths is designed as shown in Figure 1.

Overall, the array is in a T/R coil structure with etched coils on polyimide film, using flexible printed circuit board technology, comprising a large uniform exciting coil and 64 sensing coil elements, and has a spatial resolution of 0.8 mm and an effective coverage length of more than 50 mm. The sensor array has a four-layer structure, where the exciting coil is configured on the top and bottom layers and the sensing coils on two middle layers. Every coil is composed of two parts in different layers, which are connected in series through vias, respectively. The flexible design of the sensor resolves the problem of complex surface detection, and allows compact contact between sensors and materials. The two-layered exciting coil is designed in a periodic structure, where a racetrack-like loop coaxial disposition with the sensing coil elements forming a single period. The main design features and advantages of the sensor array are as follows:

1. Redundant dummy elements are designed on both sides of the array to maintain the periodic structure, eliminate the influence of fringe effect, and ensure the consistency of array elements (labeled “1” in Figure 1c);
2. Because of the flexible printed circuit board technology, the array has very high spatial resolution, thus creating favorable conditions for quantitative detection;
3. The multilayer structure design maximizes the mutual inductance between sensing and exciting coils, thus improving the response of sensing elements, and thereby increases the signal-to-noise ratio;
4. Because of the closed form and very small width of the exciting coil, defect sensitivity can be achieved in any direction with almost no blind zones, thus ensuring the detection efficiency and reliability;
5. Lead portions of the exciting and sensing coils are all designed as upper-lower parallel wires, thereby eliminating the generation of non-periodic excited magnetic field and coupled magnetic field interference introduced by sensing coil lead portions (labeled “2, 3”);
6. 3D finite element simulation can be simplified by exploiting the periodicity of the sensor, with which the response of the sensor can be predicted;
7. Copper grid wires are clad on both sides of the array to make the surface smoother, and improve the reliability of contact between sensor and material (labeled “4”).

## 3. Principle and Simulation

To understand the performance of the sensor, a finite element simulation is conducted on the sensor array presented in Section 2. The AC/DC module in the COMSOL multiphysics software is used for the simulation of a single period model due to its spatial periodicity. The principle of ECNDT is as follows: when an alternating current passes through the coil above the conductive material, an eddy current is generated on the conductor. The excited magnetic field and induced magnetic field are superimposed while the induced magnetic field inhibits the excited magnetic field. Sensing coils located above the material measure the superimposed magnetic fields passing through the planes of the coils, and the induced AC voltage can be calculated as shown in Equation (1):

(1) $$V_{out}=\;\frac{d\phi }{dt}=NS\;\frac{dB}{dt}$$

where, *V_out_* denotes induced AC voltage. ϕ is the magnetic flux which is the product of the number of turns of sensing coil *N*, area of sensing coil *S* and magnetic flux density *B*, and *t* is time. The AC voltage depends on several factors such as material conductivity, lift-off, magnetic permeability, geometrical discontinuity, frequency, excitation current, as well as spatial structure and size of the excitation and detection coils. Usually a transimpedance which equals the output voltage *V_out_* divided by the input current *I_in_* is used to represent the performance of a T/R eddy current sensor. As the magnetic flux density *B* relies on the amplitude and angular frequency of the exciting current, permeability, conductivity, geometric discontinuity of material and lift-of (distance between the sensor coil plane and the surface of tested material), an implicit expression can be established to depict the relation between acquired transimpedance change and the unknown parameters as shown in Equation (2):

(2) $$Z_{transimpedance}=\frac{V_{out}}{I_{in}}=f(\mu ,\sigma ,\omega ,X,l)$$

where, *Z_transimpedance_* stands for the transimpedance of the eddy current sensor, *μ* and *σ* are the permeability and conductivity of the material respectively, *ω* is the angular frequency of exciting current, *X* denotes the geometric discontinuity of material, and *l* stands for the lift-off.

Since the output voltage and input current are both AC signals, the transimpedance is a complex value which can be expressed as either a real part and imaginary part or amplitude and phase. The planar coil sensors normally work at 100 kHz–5 MHz. High frequency can boost the induction voltage of sensing coils, improve the signal-to-noise ratio, yet it increases the complexity of the electronic system.

**Figure 2:**
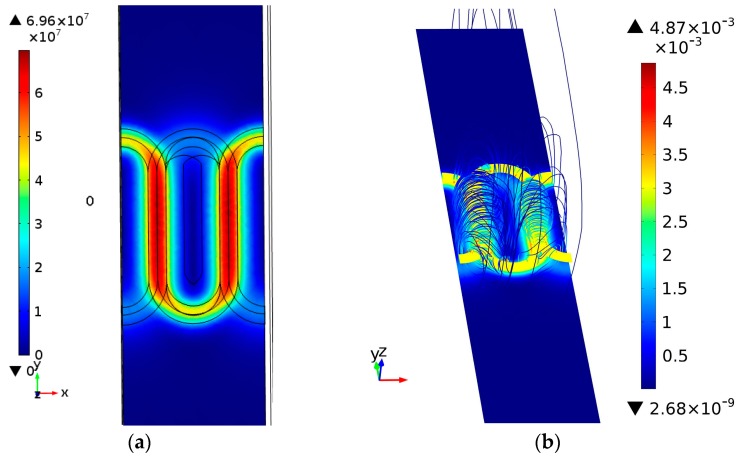
(**a**) Distribution of eddy currents; (**b**) Distribution of magnetic fields.

A single-period model is simulated with a 3 MHz sine wave excitation while the sensor is located 0.05 mm above the surface of a defect-free material with a conductivity of 18.5 MS/m (31.9% IACS). The instantaneous distribution of the magnetic field and current density of the sensor at the instant *ωt* = 0 is shown in Figure 2a. As can be seen, a racetrack-shaped eddy current loop similar to the exciting coil is produced beneath the surface of the material. Since the exciting coil is superposed by two 3/4 tracks at different layers, with overlapping in the straight portion, eddy current intensity below the straight tracks is the strongest, while it is weaker below the annular side tracks, especially on the side with larger lift-off. The eddy current response is symmetric along the YZ plane, while it is asymmetric on the XZ plane, leading to an anisotropic response of defects in different directions. When defects are perpendicular to the straight track, that is, when cracks are parallel along the *X*-axis direction, the impediment to eddy currents is greater, and therefore the array is more sensitive; conversely, when the crack length direction is parallel to the *Y*-axis, the sensitivity of the array decreases, therefore, the optimal sensitive direction of the array is the one parallel to the *X*-axis and perpendicular to the straight track direction. Quantification of crack length is also based on the optimal sensitive direction. Since the sensor array is anisotropic, the directions of cracks are determinable, thereby achieving optimal sensitive detection. Figure 2b shows the spatial distribution of magnetic fields generated by the sensor array. As can be seen, the flux almost passes through the sensing coil, which means a high mutual inductance and sensitivity are obtained.

## 4. Crack Length Sizing Algorithm

**Figure 3:**
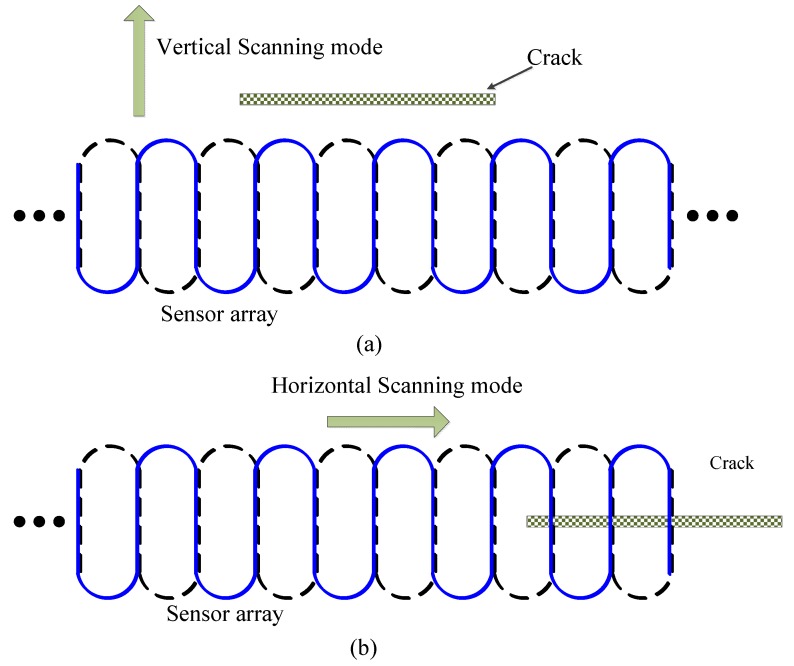
Two scanning modes: (**a**) V mode; (**b**) H mode.

Before determining the quantitative detection method, the characteristics of the sensor response to defects are studied through simulation and experiment. As stated earlier, the goal of this paper is to design a flexible sensor array, and to quantify the length of microcracks. In fact, length, width and depth of cracks all impact the sensor response. The influences of width and depth on sensor response should be studied first, and then the correlation between sensor response and crack length is investigated. Two scanning modes, *i.e.*, vertical scanning mode (V mode) and horizontal scanning mode (H mode), are employed during the simulation to study the correlation of sensor response with crack size as shown in Figure 3. V mode means the scanning direction is perpendicular to the crack length direction on the optimal sensitivity direction premise, while H mode means the scanning direction is parallel to the crack length direction on the optimal sensitivity direction premise with the crack right below the sensor array.

### 4.1. Effect Analysis of Crack Width

**Figure 4:**
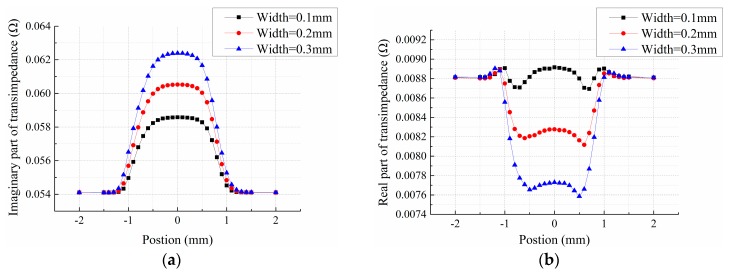
(**a**) Imaginary part; (**b**) Real part. Of sensor transimpedance in vertical scanning mode for cracks with different width.

On the basis of the model shown in Figure 2, the lift-off is set to be 0.05 mm, 1 mm-deep cracks with varying widths are added and the central location of cracks are altered to obtain the sensor transimpedance in V scanning mode. Due to its periodic boundary conditions, crack length is set to be infinitely long in this case, and the response of the sensing element above the center of a crack is acquired. The relationship between the imaginary and real parts of the sensor transimpedance *versus* the crack position is shown in Figure 4a,b, respectively. It shows that the transimpedance output curves of both the imaginary and real parts present a single peak, where the imaginary part presents a crest curve and the real part exhibits a trough curve. Nevertheless, compared to the imaginary part, the real part is much smaller and the signal-to-noise ratio is poorer. Moreover, as the essence of an eddy current sensor, mutual inductance is mainly manifested in the imaginary part of the transimpedance. Therefore, in the subsequent simulation and experiments, the crack response curve of the transimpedance imaginary part is mainly studied. As seen from Figure 4a, different crack width influences the peak value of the response curve. As crack width grows, the peak value increases. However, the waveforms are similar. Defect response begins to rise when the crack is near the bottom edge of the exciting coil, reaches a climax when the crack center coincides with the center of the sensing coils, and ends when crack leaves the other edge of exciting coil, suggesting that the sensor array is only sensitive to the coverage area, while insensitive to the peripheral areas. Thus, the sensor array can be used for measuring the discontinuous change of material properties and detecting edge defects, which are precisely the insufficiencies of the conventional eddy current sensors. Crack response is not centrosymmetric, and this is associated with the aforementioned two-layered structure of exciting coil. Based on the above analysis, the influence of crack width can be eliminated by normalization processing of the crack response, thus the transimpedance data are uncorrelated with the crack width.

### 4.2. Effect Analysis of Crack Depth

**Figure 5:**
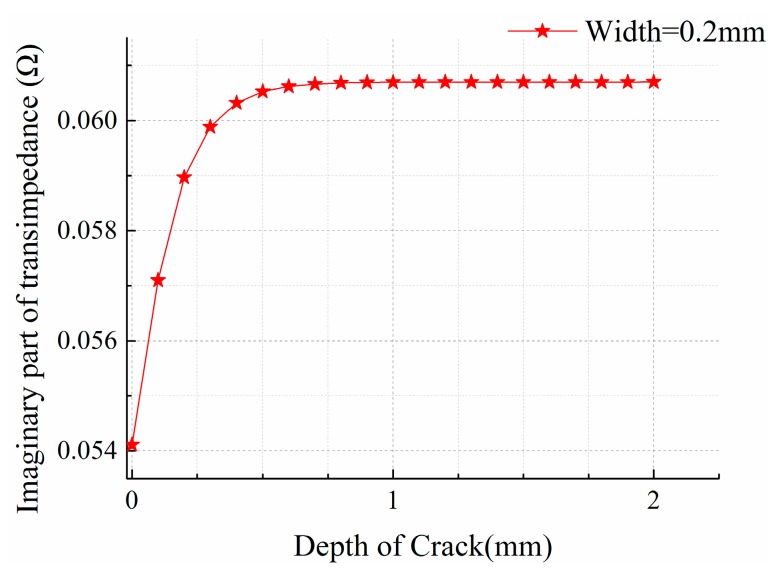
Sensor response of cracks with different depth.

Next the influence of crack depth on transimpedance was studied. Crack width and lift-off are fixed at 0.2 mm and 0.05 mm, respectively, while the depth varies between 0–2 mm in 0.1 mm intervals. Cracks are located beneath the center of the sensing element, where the sensing element has maximum output. The graph of transimpedance imaginary part *versus* crack depth is shown in Figure 5. 

As can be seen, the imaginary part of the transimpedance increases rapidly at the beginning and then gradually slows down with increasing crack depth. When the crack depth reaches 1 mm, the transimpedance stabilizes. The following corollary can be derived: the sensor array has high sensitivity to small shallow defects, which can detect only 0.1 mm deep cracks; when the crack depth exceeds 1 mm, the depth becomes irrelevant to the crack response signals. The reason is that when the sensors work under a 3 MHz excitation, the eddy current is concentrated on the surface of material, hence, even very shallow surface cracks cause great changes in transimpedance. For shallow cracks, the eddy currents tend to bypass them from the bottom of crack, and when cracks are deeper than 1 mm, the eddy current will be blocked up and not able to pass up from the bottom of the crack. Crack impediment on eddy current is consistent, so the response does not change along with crack depth.

### 4.3. Quantitative Analysis of Crack Length

**Figure 6:**
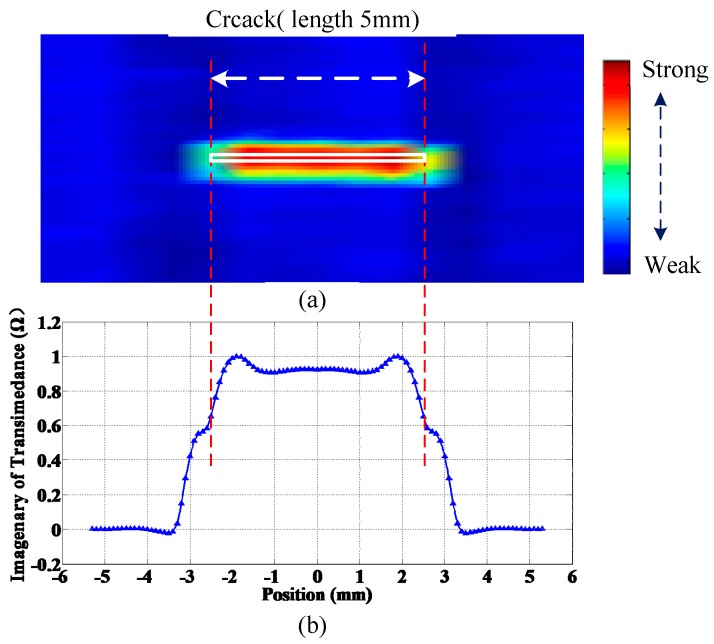
(**a**) C-scan image of a 5 × 0.2 × 0.5 mm defect; (**b**) Simulated peak output curve along length direction.

Figure 6a shows a C-scan image of a crack together with the position, whose size (length × width × depth) is 5 × 0.2 × 0.5 mm, while the normalized response of the crack in H mode scanning is simulated and shown in Figure 6b. 

As can be seen, the crack signal is substantially similar to an isosceles trapezoid, and the width of the trapezoid is closely related to the length of the crack. The longer the crack is, the wider the trapezoid becomes. It can be seen that the two beveled edges of the trapezoid are not inerratic lines, with inflection points in the middle, which correspond to edge of the crack, thus the quantification of crack length lies in the identification of crack edge location. One simple length estimation method is to set a threshold, that is, when the difference between the element response and the response signal in the absence of defects exceeds a certain value, the element is confirmed to be located above the crack, and hence, crack length can be obtained by multiplying the number of crack response elements by the spatial resolution. Other main crack size reconstruction methods are the prebuilt crack response database-based neural network, optimal problem-solving method, *etc.* [7]. These approaches require simulation of variously sized cracks in advance and calculation of crack response signals, which bring about a significant computational burden; besides, accuracy depends on the database size and the matching algorithm. What is more, the length of the response signal is 6.8 mm *versus* a real crack length of 5 mm. This means that the response of a single element is sensitive to not only the area below the element, but also the area below the adjacent element. In actual inspection, due to the limited spatial resolution of the array, which is 0.8 mm, the measured crack response curve is equivalent to the sampling on a continuous curve at a 0.8 mm interval. The crack length sizing method proposed in this paper is named crack edge determination method using simulation based normalized crack signal fitting (NCSF). The principle of the NCSF method is to pinpoint the location of crack edges through fitting the discrete measured data derived from the sensor array based on the normalized simulated crack signals. By embedding the fitting algorithm into the real-time scanning imaging software, and combining with an encoder, positioning, real-time imaging and length sizing of cracks can be achieved synchronously.

## 5. Experiments and Discussion

### 5.1. Experimental System Setup

**Figure 7:**
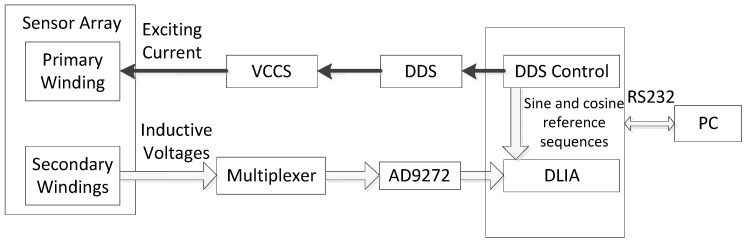
Schematic diagram of the measurement system.

Signal processing of the sensor array is achieved with a multi-channel transimpedance measurement system, which has been described in earlier work [17]. A block diagram of the measurement system is shown in Figure 7. Fast measurement of 64-channel transimpedance is achieved based on FPGA and AD9272 with multiplexers. Transimpedance data is calculated by FPGA and transmitted to the computer via a serial port; then data calibration, 3D real-time imaging, crack localization and automatic crack length sizing are further completed by the computer.

### 5.2. Experimental Verification

**Figure 8:**
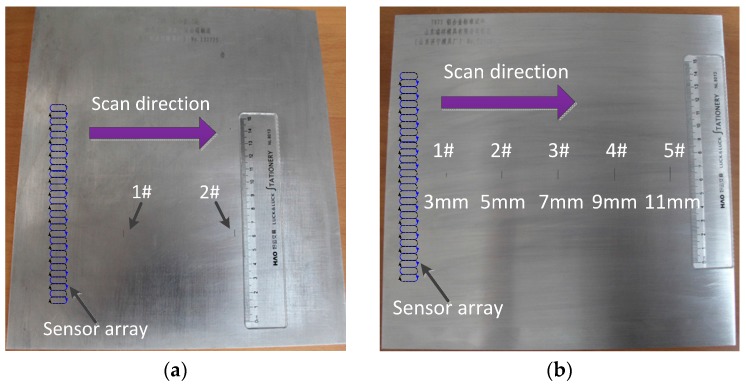
(**a**) Specimen 1 with cracks of different width; (**b**) Specimen 2 with cracks of different length.

To verify the reliability of the simulation results and the validity of the NCSF algorithm, two standard specimens machined with different sized cracks are tested, as shown in Figure 8. The specimens are produced by Shandong Ruixiang Mould Ltd. (Jining, China), which is a professional manufacturer of NDT testing specimens, and a member of National Technical Committee on Non-destructive Testing of Standardization Administration of China. Two specimens are both made of 7075 aluminum alloy, with an actual conductivity of 18.5 MS/m, which is measured with a SIGMATEST 2.069 US instrument (Foerster Instruments, Pittsburgh, PA, USA). 

**Figure 9:**
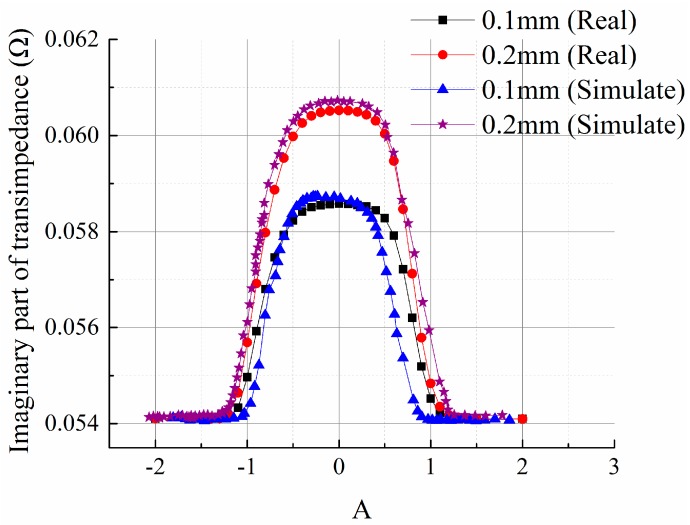
Output curve of transimpedance imaginary part under vertical scan mode.

The size of specimen 1 is 250 × 250 × 20 mm, two different wide cracks sizing 5 × 0.1 × 1 mm and 5 × 0.2 × 1 mm are machined on its surface, which are marked as crack 1 and crack 2 respectively, as shown in Figure 8a. The size of specimen 2 is 250 × 250 × 10 mm, and five cracks whose lengths ranging from 3 mm to 11 mm are machined on it, as is shown in Figure 8b. The five cracks on the surface of specimen 2 have the same width (0.2 mm) and depth (1 mm). Specimen 1 is firstly inspected under vertical scanning mode and the transimpedance output curve of the sensing element passing over the center of the crack is obtained and shown in Figure 9. As can be seen, the experimentally measured curves are basically consistent with the simulation results. Crack width influences the peak of the output curve. The larger the width is, the greater the peak will be.

**Figure 10:**
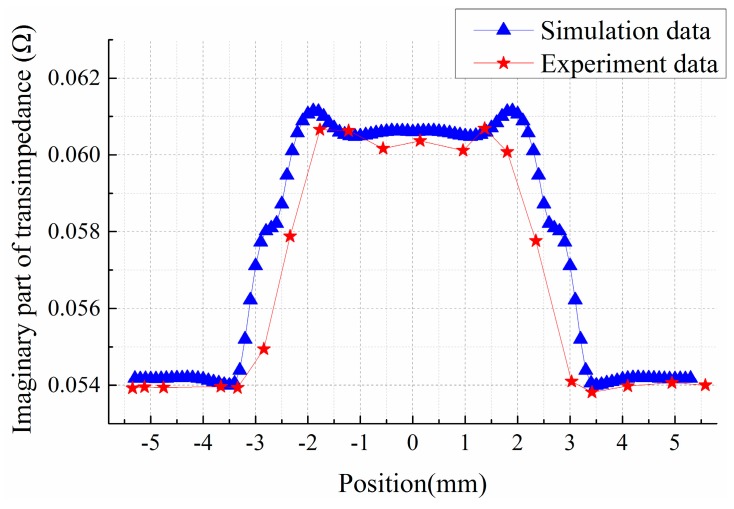
Response of crack 2 in H mode scanning.

The response of crack 2 on specimen 1 under H mode scanning is shown in Figure 10. As can be seen, the experimental result is basically consistent with the simulation result. It is also a trapezoid structure, peaking around the two apical angles and flat in the middle. Due to the few sampling points, non-linear curves cannot be reflected at the rising and falling edges on both sides. This is because the actual scanning is done manually without assistance of any scanning mechanism, so the experimental results fail to reflect all the details. However the comparison of the results reveals almost identical width of crack signals, which further verifies the validity of the analysis of the spatial distribution of crack response.

### 5.3. Quantitative Experiment

#### 5.3.1. Planar Objects Testing

A crack length sizing experiment is done to validate the NCSF algorithm. Firstly, two defects on specimen 1 are tested separately and repeatedly, and the results are shown in Table 1. As can be seen, the algorithm is highly precise in measuring the length of cracks with different widths. The average measured crack length is around 4.94 mm for crack 1, and around 5.08 mm for crack 2, with measurement accuracy approximating ±0.2 mm. The experimental results demonstrate that the NCSF algorithm is effective for cracks of different widths.

**Table 1:** Crack length sizing results of specimen 1.

Times	#1 (mm)	#2 (mm)
1	4.92	5.04
2	4.95	5.12
3	4.86	4.98
4	5.08	5.06
5	5.01	5.16

Cracks with different length but same width and depth (shown in Figure 8b) are tested too. The crack length sizing algorithm based on threshold and the NCSF algorithm are both applied separately for quantitative measurement of actual cracks, and the results are shown in Table 2. Every crack is measured and recorded three times. The results show that algorithm based on threshold is able to size the crack length, but not accurately, with resolution equaling a spatial resolution of 0.8 mm, and error of ±0.8 mm. While using the NCSF algorithm, crack length sizing accuracy increases, presenting a measurement accuracy of ±0.2 mm. These results indicate that the optimized flexible sensor array designed herein has a high sensitivity to microcracks, and a sizing accuracy within ±0.2 mm is obtained using the proposed NCSF algorithm.

**Table 2:** Quantitative results of specimen 2.

Crack Number	Threshold Based Algorithm (mm)			NCSF Algorithm (mm)		
	First Time	Second Time	Third Time	First Time	Second Time	Third Time
1	2.4	3.2	2.4	3.08	3.14	2.95
2	4.8	5.6	4.8	4.89	5.06	5.1
3	6.4	7.2	7.2	7.02	7.09	7.1
4	8.8	8.8	9.6	9.05	9.11	8.86
5	10.4	11.2	11.2	11.18	11.02	11.15

#### 5.3.2. Curved Surface Testing

**Figure 11:**
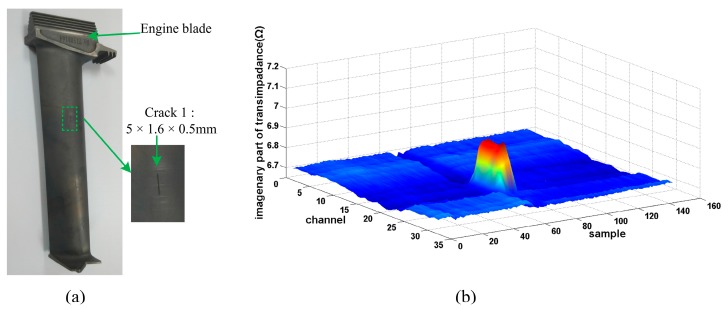
Scanning of engine blade. (**a**) Engine blade; (**b**) Image of crack.

An aircraft engine blade with complicated geometry was scanned. An EDM crack with 5 mm length was measured. Thanks to the flexible probe design, the crack is detected well and the length is also measured. A C-Scan of the crack is shown in Figure 11. It is evident that the crack is scanned with good SNR. Therefore, the flexible array probe is suitable for objects with complicated geometry as well as crack length sizing.

#### 5.3.3. Fatigue Crack Testing

**Figure 12:**
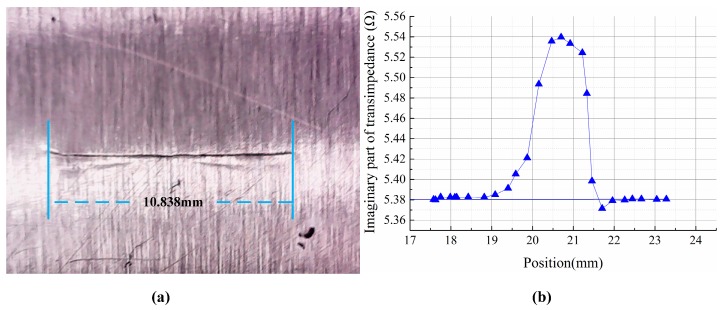
(**a**) Specimen with fatigue crack; (**b**) Output of single element under V mode scanning.

A fatigue crack on an aluminum plate which is produced by a tension and compression fatigue testing machine is also scanned. Figure 12 shows the specimen and the result. The size of the crack is 10.838 mm (length) × 0.042 mm (width). As can be seen from Figure 12b, due to the small width of the fatigue crack, the peak value is relatively low compared to the EDM crack signal. Both quantitative results of engine blade and fatigue crack are listed in Table 3. As is shown in the table, the result is a little smaller than the true value. This is probably because the fatigue crack is not in a straight line. There is a segment stray from the crack line at the left corner, shown in Figure 12a.

**Table 3:** Quantitative results of blade crack and fatigue crack.

Times	Blade Crack (mm)	Fatigue Crack (mm)
1	4.85	10.80
2	4.82	10.69
3	4.96	10.52
4	5.08	10.93
5	5.01	10.65

## 6. Conclusions

A novel flexible eddy current sensor array for quantitative measurement of micro fatigue cracks in key components of aircraft, *etc.* is designed in this paper. The flexible design conforms the sensor to the curved surface of engine blade. The principal of the sensor array and the response of cracks with different sizes are demonstrated by simulation. The NCSF algorithm is developed to size crack length with high accuracy. Experimental results are proved consistent with simulations. All results prove that the array is not only sensitive to microcracks, but also capable of crack length sizing. It has the following advantages:

1. Flexible, and capable of inspecting complex geometric structures;
2. Sensitive to microcracks, and capable of crack length sizing, with an accuracy within ±0.2 mm;
3. High spatial resolution, reaching 0.8 mm; array element of 64 mm, covering a length of over 50 mm; high detection efficiency; sensitive to microcracks with different directions.
